# Molecular mechanism underlying *Oleum Cinnamomi*-induced ferroptosis in MRSE via covalent modification of AhpC

**DOI:** 10.3389/fphar.2025.1554294

**Published:** 2025-07-22

**Authors:** Jianchao Wang, Ziqi Wu, Qianying Chen, Danna Yan, Yuanqiang Ling, Yuan He, Lu Jin, Guomin Zhao, Huayong Peng, Depo Yang

**Affiliations:** 1 School of Pharmaceutical Sciences, Jishou University, Jishou, China; 2 School of Pharmaceutical Sciences, Sun Yat-sen University, Guangzhou, China; 3 Guangdong L-Med Biotechnology Co., Ltd, Guangzhou, China; 4 Zhongshan Institute for Drug Discovery, Shanghai Institute of Materia Medica, Chinese Academy of Sciences, Zhongshan, China

**Keywords:** Oleum Cinnamomi (OC), MRSE, covalent inhibitors, AhpC, ROS, metabolic pathways

## Abstract

**Introduction:**

*Oleum Cinnamomi* (OC) is a volatile oil extracted by steam distillation from the dried branches and leaves of *Cinnamomum cassia* Presl, a plant belonging to the Lauraceae family. For centuries, OC has been utilized as a food preservative and flavoring agent, demonstrating potent inhibitory effects against bacteria and fungi. It is particularly effective in controlling infections caused by Methicillin-Resistant *Staphylococcus epidermidis* (MRSE), which often parasitizes the skin surface. To uncover the target and molecular mechanism by which OC eradicates MRSE, this study initially assessed the impact of OC and its primary constituents on oxidative stress in MRSE cells.

**Methods:**

Mass spectrometry was employed to identify the target and covalent binding sites of OC, while a kit was used to monitor changes in key biomolecules of MRSE cells exposed to OC. Additionally, the efficacy of OC in inhibiting MRSE adhesion and infection of RAW 264.7 mouse macrophages was evaluated.

**Results:**

The findings revealed that OC’s main components, cinnamaldehyde and 2-methoxycinnamaldehyde, covalently modify MRSE and AhpC. This modification disrupts the AhpC-AhpE regeneration cycle, thereby disturbing both enzymatic and non-enzymatic redox homeostasis. It leads to intracellular ROS accumulation and effectively prevents MRSE from adhering to RAW 264.7 mouse macrophages. In response to ROS detoxification, MRSE attempts to upregulate the expression of TCA cycle-related proteins. However, the continuous accumulation of ROS inactivates the [Fe-S] protein of Aconase (ACO), hindering ACO’s catalytic conversion of citric acid to isocitrate. This results in sustained intracellular accumulation of citric acid, limiting the TCA cycle and ATP generation. Simultaneously, enzymes involved in reduction catalysis, such as superoxide dismutase (SOD), peroxidase reductase (Prx), and glutathione synthase (GCL), are collectively inactivated. OC induces oxidative stress in MRSE, depleting GSH and triggering lipid peroxidation, which in turn induces MRSE to undergo ferroptosis.

**Discussion:**

This covalent inhibition strategy targeting AhpC to induce ferroptosis offers a promising approach for effectively treating and preventing MRSE infections, thereby opening new avenues for combating drug-resistant pathogen infections.

## 1 Introduction

The increasing prevalence and dissemination of multidrug-resistant (MDR) pathogens, such as Methicillin-Resistant *Staphylococcus aureus* (MRSA), Multidrug-Resistant *Pseudomonas aeruginosa* (MRPA), and vancomycin-resistant *Enterococcus faecium*, have emerged as a formidable global health crisis (Song et al., 2021). Among these, *Staphylococcus epidermidis*, a frequent colonizer of hospitalized and immunocompromised individuals, is also progressively acquiring multidrug resistance. A study investigating orthopedic implant infections reported that over 80% of 342 clinical isolates of *Staphylococcus epidermidis* exhibited resistance to penicillin, ampicillin, cefazolin, and cefamandole, underscoring the escalating threat posed by methicillin-resistant *Staphylococcus epidermidis* (MRSE) (Hou et al., 2013). The dwindling arsenal of effective antibiotics, coupled with the unchecked proliferation of MRSE infections, poses a significant risk to the health and wellbeing of vulnerable populations.

With the escalating challenge of managing multidrug-resistant bacterial infections in clinical settings, there is an urgent need for researchers to identify novel antimicrobial agents or develop innovative strategies to counteract bacterial evolution and resistance. Historically, natural products and their derivatives have been a vital source of new antibiotics (Liao et al., 2022; Singh et al., 2024). Among these, *Cinnamomum cassia* Presl has shown promising efficacy against resistant pathogens, including MRSE, MRSA, and MRPA. The active metabolites of *Cinnamomum cassia* Presl possess notable antimicrobial properties, particularly against drug-resistant bacteria. For example, cinnamaldehyde (CA), a metabolite of *Cinnamomum cassia* Presl, displays broad-spectrum antimicrobial activity, effectively inhibiting a range of pathogens such as *Staphylococcus aureus*, MRSA, *Streptococcus pneumoniae*, *Escherichia coli*, and *Listeria monocytogenes*, while also disrupting biofilm formation (Utchariyakiat et al., 2016). Our previous research has demonstrated that 4-methoxy cinnamaldehyde (MCA), another metabolite of *Cinnamomum cassia* Presl, significantly elevates reactive oxygen species (ROS) levels in MRSE, thereby disrupting amino acid and energy metabolism and inhibiting bacterial proliferation (Qian et al., 2022).

*Oleum Cinnamomi* (OC) is a complex mixture primarily composed of CA that is extracted from *Cinnamomum cassia* Presl (Peng et al., 2024). OC has a long history of use in traditional Chinese medicine and is included in the 2020 edition of the Chinese Pharmacopoeia (Pharmacopoeia, 2020). When applied topically, OC exhibits potent antibacterial and antipruritic effects, particularly against *Staphylococcus epidermidis*. Moreover, OC demonstrates antimicrobial activity against various bacteria, including *Escherichia coli*, *Staphylococcus aureus*, and *Salmonella enteritidis*, and inhibits the growth of fungi such as *Candida albicans* and *Aspergillus niger* (Vasconcelos et al., 2018; Zhang et al., 2016). OC is also widely used in India as a preservative to prevent yeast spoilage. These multifaceted properties render OC a promising candidate for applications in both the food industry and medical fields. Despite its notable inhibitory effects on bacteria and fungi, the molecular targets and mechanisms underlying OC’s action against *Staphylococcus epidermidis* remain largely unexplored (Zhang et al., 2016). To address this gap, the present study employs advanced techniques such as chromatography-mass spectrometry to elucidate the molecular targets and potential mechanisms of OC against MRSE. Findings from this research may provide valuable insights and pave the way for developing novel strategies to combat multidrug-resistant bacterial infections.

## 2 Materials and methods

### 2.1 Materials and reagents

#### 2.1.1 Cell lines

MRSE (RP62A/ATCC 35984) was purchased from the Guangdong Microbial Culture Collection Center and purified using the streak plate method. The Raw 264.7 cell line is from the American Type Culture Collection (ATCC).

#### 2.1.2 Reagents

CA, MCA, cefazolin, amoxicillin, vancomycin hydrochloride, gentamicin sulfate, and clindamycin hydrochloride were of analytical grade. The NAD^+^/NADH Quantification Kit (Bioss, AK300). Reduced Glutathione Detection Kit (Solarbio, BC1175), the GPX ELISA Kit (Enzyme-Linked, MI035800). The llipid peroxidation (LPO) Content Detection Kit (Solarbio, BC5240). The SOD Detection Kit (Beibo, BB-47105), the Prx ELISA Kit (Xinsemei, CSM-925924O2). The Chemiluminescence ATP Content Detection Kit (G4309, Sevier Biotech). CAMHB broth medium and M-H solid agar medium (Guangdong Huankai Microbial Technology Co., Ltd.), FBS serum and DMEM medium (Sigma-Aldrich), HBSS medium (Savil Biotech Co., Ltd.), PBS buffer (Guangzhou Huada Gene Technology Co., Ltd.), DCFH-DA ROS fluorescence probe (Beijing Solarbio Science & Technology Co., Ltd.), and DCFH-DA fluorescence dye (Montclair, California, United States, Biotech Company).

#### 2.1.3 Instruments

T6 New Century UV-Vis Spectrophotometer (Beijing Purkinje General Instrument Co., Ltd.), Heratherm™ Microbial Incubator (Thermo Fisher Scientific, Germany), ECO Ultra-clean Workbench (Thermo Fisher Scientific, Germany), LDZX-50FBS High-Pressure Steam Sterilizer (Shanghai Minquan Instrument Co., Ltd.), BSI-3C Constant Temperature Shaker (Shanghai Beiying Industrial Co., Ltd.), EVOS M700 Integrated Cell Imaging and Analysis System (Thermo Fisher Scientific, Germany), NanoLC-ESI MS/MS (Waters Corporation, United States), Synergy H1 Multifunctional Microplate Reader (BioTek Instruments, United States).

### 2.2 Experimental procedures

**FIGURE 1:**
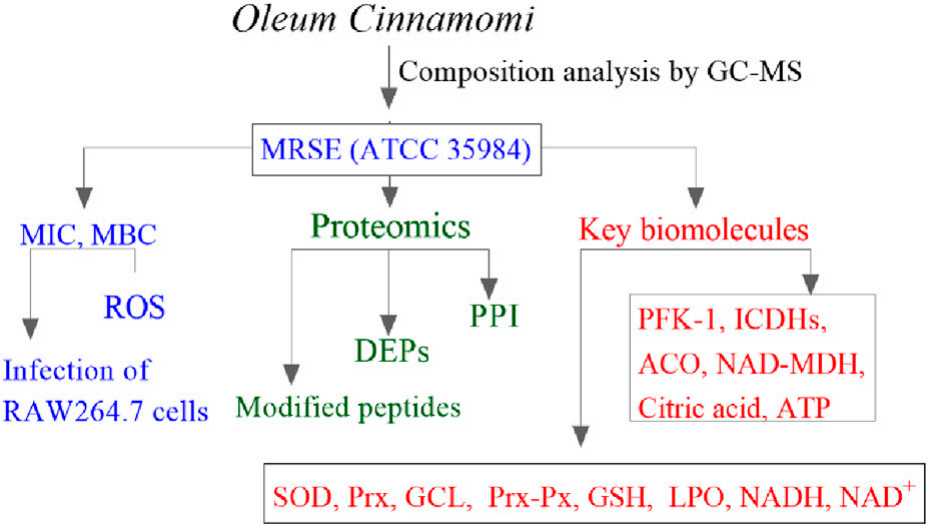
The experimental flowchart in this study. MIC, minimum inhibitory concentration; MBC, minimum bactericidal concentration; ROS, reactive oxygen species; DEPs, differentially expressed proteins; PPI, protein–protein interaction; PFK-1, phosphofructokinase-1; ICDHs, isocitrate dehydrogenases; ACO, aconitase; NAD-MDH, NAD-dependent malate dehydrogenase; SOD, superoxide dismutase; Prx, peroxiredoxin; GCL, glutamate-cysteine ligase; Prx-Px, peroxiredoxin-peroxidase; GSH, glutathione; LPO, lipid peroxidation; NADH/NAD^+^, nicotinamide adenine dinucleotide.

Experimental procedures in this study were conducted in accordance with the flowhart illustrated in Figure 1.

### 2.3 Preparation of OC from *Cinnamomum cassia* presl

In mid-May 2023, fresh branches and leaves of *Cinnamomum cassia* Presl [Lauraceae; Cinnamomi cassiae cortex] were procured from Luoding District, Zhaoqing City, Guangdong Province, China. The authenticity of the plant was verified by Professor Yang Depo from Sun Yat-sen University and identified as Cinnamomum cassia Presl based on morphology. A specimen with the identification number 20231125004 is preserved at the Medicinal Herb Display Center of Sun Yat-sen University. The plant materials were then dried at room temperature in a shaded area, away from direct sunlight. The dried plant materials were cut up, ground, and subsequently passed through a 60-mesh sieve. A total of 150 g of *Cinnamomum cassia* Presl powder was placed in a 2000 mL round-bottom flask, to which 1,000 mL of distilled water and some zeolites were added. The mixture was then subjected to steam distillation for 4 h at 100 °C using a Clevenger apparatus. Following distillation, the resulting oil was collected and dehydrated with anhydrous sodium sulfate for 24 h. The quantity of the obtained oil was measured three times and expressed per mass of the dry plant material. The yield of the oil was 0.33% (w/w). The resulting yellow clear liquid, characterized by its distinctive odor, was stored at 4 °C in an amber vial for subsequent analysis. The relative density is 1.059 and the refractive index is 1.608. The composition of the oil was identified using GC-MS. The Pharmacopoeia stipulates that the content of cinnamaldehyde determined by gas chromatography shall not be less than 75.0%. The cinnamaldehyde content of this extract is 89.32%, which complies with the Pharmacopoeia requirements (Pharmacopoeia, 2020).

### 2.4 Antibacterial susceptibility testing

According to the 2017 guidelines of the Clinical and Laboratory Standards Institute the minimum inhibitory concentration (MIC) and minimum bactericidal concentration (MBC) were determined by broth microdilution to assess the antimicrobial activity (Chen et al., 2022). Briefly, 100 µL of bacterial suspension diluted to 2 × 10^6^ CFU/mL was inoculated into a 96-well plate and treated with different concentrations of the drug, with 0.1% DMSO as the vehicle control. After incubation at 37°C for 24 h, the optical density (OD) at 600 nm (OD_600_) was measured using a microplate reader. The minimum concentration at which OD_600_ < 0.1 was defined as the MIC.

### 2.5 Evaluation of ROS generation mediated by OC

The ROS levels in bacteria after MCA treatment were measured using the CM-H_2_DCFDA staining method described in the literature (Nabavi et al., 2015). The logarithmic-phase MRSE was resuspended to an OD_600_ of 0.2 and incubated in CAM medium containing OC (1×MIC, 2×MIC) at 37°C with shaking for 4 h. The bacteria were collected by centrifugation at 4°C, washed twice with PBS, and then adjusted to an OD_600_ of 0.2. The bacteria were stained with 2 µM CM-H_2_DCFDA at 37°C for 45 min. A 0.1% DMSO treatment group was used as the control. After washing with HBSS, the bacterial suspension was analyzed by flow cytometry, and the median fluorescence intensity (MFI) was quantified.

### 2.6 Evaluation of OC synergistic effect with RAW264.7 cells in killing MRSE

#### 2.6.1 Fluorescent labeling of MRSE treated with OC

Logarithmic-phase MRSE was treated with 1×MIC or 2×MIC of OC and incubated at 37°C with shaking for 4 h. The bacteria were collected by centrifugation at 4°C and resuspended in PBS, adjusted to a final volume of 5.0 mL (OD_600_ = 0.2). CFDA-SE staining solution was added to a final concentration of 2 μM, and the bacteria were incubated at 37°C for 20 min. After centrifugation at 4°C, the bacteria were washed three times with HBSS containing 1% FBS, and the final volume was adjusted to 1 mL. The bacterial suspension was stored at 4°C and used within 2 h (Qian et al., 2022).

#### 2.6.2 Measurement of MRSE infection in RAW264.7 cells

RAW264.7 cells (5 × 10^6^ cells/well) were seeded in fresh DMEM medium containing 10% fetal bovine serum and incubated at 37°C for 12 h in a humidified atmosphere with 5% CO_2_. After removing the culture medium, the cells were gently washed with HBSS containing 1% FBS. CFDA-SE-labeled MRSE suspension (MOI = 2.5:1) was added to the cells and incubated at 37°C for 2 h. After incubation, the cells were gently washed three times with HBSS containing 1% FBS. Fluorescence microscopy was used to observe MRSE adhesion to macrophages using the green fluorescence channel suitable for CFDA-SE dye. The cells were then collected, centrifuged at 1,000g for 3 min, and the supernatant was discarded. The cells were washed twice with HBSS containing 1% FBS. Flow cytometry was used to record the changes in the number of MRSE-infected RAW264.7 cells before and after treatment (Jin et al., 2025).

#### 2.6.3 Measurement of OC synergistic effect with RAW264.7 cells in MRSE clearance

Under the same conditions, MRSE-infected RAW264.7 cells were incubated for 2 h. After centrifugation, the cells were washed with PBS. Then, 200 µL of 0.25% trypsin solution was added, and the cells were incubated at 37°C for 15 min. After repeated pipetting to fully lyse the cells, the lysed samples were spread onto Muller-Hinton agar plates using serial dilutions. The plates were incubated at 37°C for 24 h. After incubation, the number of colonies was recorded. The percentage of viable MRSE cells and RAW264.7 cells was calculated based on the colony count, with each sample repeated at least three times.

### 2.7 Proteomics experiments

#### 2.7.1 Sample preparation for proteomics

MRSE was treated with 1 × MIC of OC for 4 h. After centrifugation, the bacterial pellets were washed twice with pre-cooled PBS, and then protein lysis buffer was added to both the 1 × MIC and control group samples. Cells were disrupted using an ultrasonic system. After centrifugation, the supernatants were measured for protein content using the BCA protein assay kit to verify extraction efficiency. The extracted proteins were digested with trypsin, desalinated, freeze-dried, and then dissolved in 40 µL of 0.1% formic acid. After centrifugation, the supernatants were analyzed by mass spectrometry. The entire process was repeated three times (Tang et al., 2020).

#### 2.7.2 NanoUPLC-ESI MS/MS determination

Peptide mixtures were separated using the Easy NanoUPLC 1,200 system (Thermo Scientific, San Jose, CA) equipped with a reverse-phase ReproSil-Pur C18-AQ column (75 μm × 30 cm, 2 μm) under gradient elution at a flow rate of 200 nL/min for 120 min. A loading quantity of 2 µL was used. Elution was performed with a gradient of 0.1% formic acid and 80% acetonitrile. The peptide segments were identified using a Q-Exactive Plus mass spectrometer (Thermo Scientific, San Jose, CA). The raw data were processed using Proteome Discoverer software (version 2.2.0.388, Thermo Fisher Scientific). The *S. epidermidis* species protein database containing 1,220 proteins was downloaded from the UniProt database (8 Jan 2024).

#### 2.7.3 Analysis of modified peptides

CA or MCA-modified peptides were identified using Proteome Discoverer software (version 2.2.0.388, Thermo Fisher Scientific) by searching the data files against the MRSE (ATCC 35984) protein database (8 Jan 2024). All searches included a 1% false discovery rate, and all modified peptides were manually checked to verify the peptide sequences.

### 2.8 Measurement of related substance levels and enzyme activity

To assess the redox homeostasis in MRSE, the following methods were used in combination with kits as referenced by (Peng et al., 2024). Intracellular NAD^+^/NADH content, Reduced glutathione (GSH) content, ATP content, Glutathione Peroxidase (GPX) activity, lipid peroxidation (LPO) content, Total Superoxide Dismutase (SOD) activity, Peroxiredoxin (Prx) activity were determined using commercial assay kits.

Statistical analysis: Unless otherwise specified, all statistical analyses were conducted in GraphPad Prism. All the experiments are in triplicate, with at least three biological replicates. The data were analyzed by one-way analysis of variance (ANOVA) and expressed as the mean ± standard error of the mean (mean ± SEM). p ≤ 0.05 were regarded to be significant (∗p ≤ 0.05; ∗∗p ≤ 0.01; ∗∗∗p ≤ 0.001; ∗∗∗∗p ≤ 0.0001 vs. control group).

## 3 Results

### 3.1 Identification of chemical components in *Oleum Cinnamomi*

**FIGURE 2:**
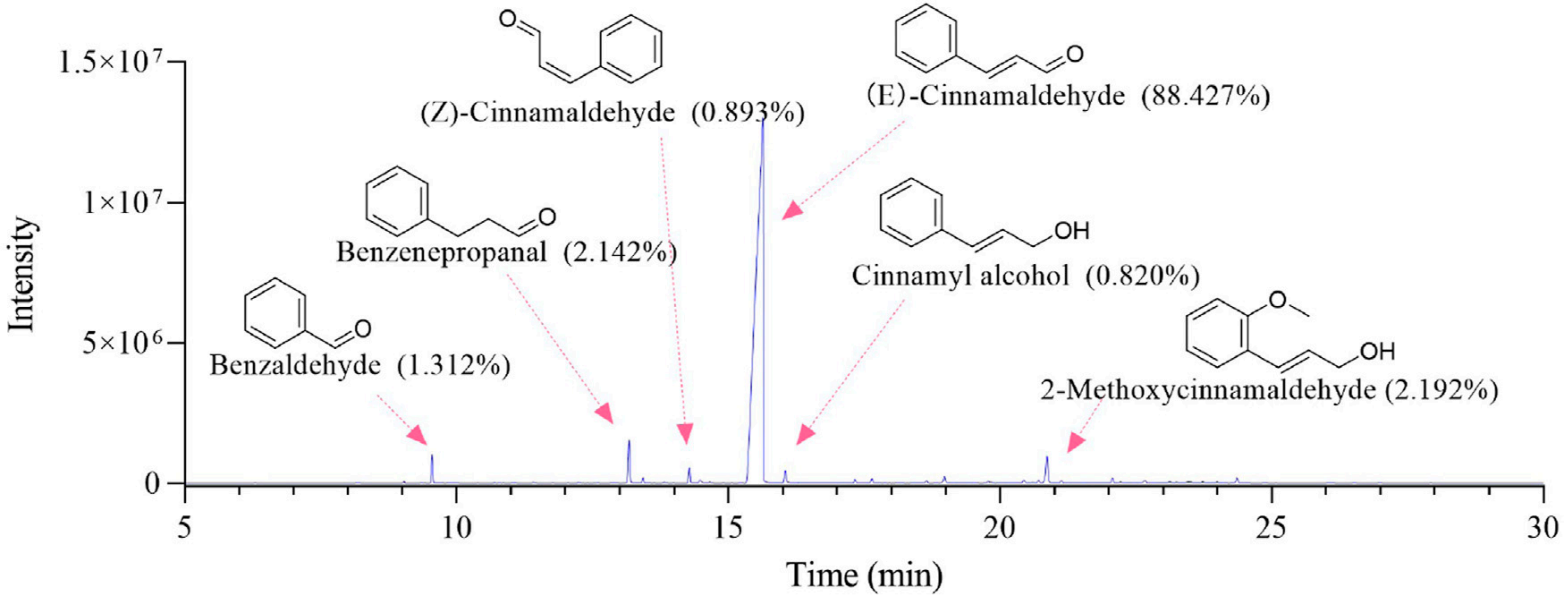
Typical total ion chromatograms of *Oleum Cinnamomi* analyzed by GC-MS.

The GC-MS total ion chromatogram of *Oleum Cinnamomi* is shown in Figure 2; Table 1. There are six components with a relative content higher than 0.80%, accounting for 95.786% of *Oleum Cinnamomi*. These components mainly include Benzaldehyde (1.312%), Benzenepropanal (2.142%), (Z)-Cinnamaldehyde (0.893%), (E)-Cinnamaldehyde (88.427%), Cinnamyl alcohol (0.820%), and 2-Methoxycinnamaldehyde (2.192%).

**TABLE 1:** Main components and relative contents (%) of OC by GC–MS analysi**s**.

Peak	RT (min)	Percentage (%)	Name
1	9.551	1.312	Benzaldehyde
2	13.175	2.142	Benzenepropanal
3	13.432	0.354	Bornanol
4	14.285	0.893	(Z)-Cinnamaldehyde
5	14.480	0.321	3-Phenylpropanol
6	15.628	88.427	(E)-Cinnamaldehyde
7	16.048	0.820	Cinnamyl alcohol
8	17.330	0.229	2-Methoxyphenylacetone
9	17.644	0.282	Copaene
10	18.646	0.245	Caryophyllene
11	18.980	0.568	Cinnamyl acetate
12	19.780	0.091	γ-Muurolene
13	20.435	0.273	β-Bisabolene
14	20.713	0.224	δ-Cadinene
15	20.863	2.192	2-Methoxycinnamaldehyde
16	21.131	0.219	α-Bisabolene
17	22.072	0.388	Spathulenol
18	22.667	0.314	Tetradecanal
19	24.360	0.412	α-Bisabolol
Total		99.706	

### 3.2 Antibacterial susceptibility testing of MRSE

In accordance with the antibacterial activity evaluation method used by Qian (Qian et al., 2022), antibacterial susceptibility testing was conducted on MRSE, as presented in Table 2. The results indicated that MRSE (ATCC35984) exhibited resistance to Cephalexin, Gentamicin, and Clarithromycin, while demonstrating sensitivity to Vancomycin, with both the MIC and MBC values recorded at 1.2 μg/mL. This finding confirms that the MRSE (ATCC35984) strain utilized in this study is multidrug-resistant. The MIC of OC against MRSE was determined to be 240 μg/mL, with its principal component, CA, exhibiting an MIC of 200 μg/mL, and MCA presenting an MIC of 220 μg/mL. The antibacterial efficacy of OC against MRSE aligns with findings reported in the existing literature. Notably, the addition of vitamin C (VC) to the OC antibacterial system resulted in a significant increase in the MIC of OC against MRSE, suggesting that the reducing agent VC antagonizes the antibacterial activity of OC. Furthermore, MRSE demonstrated sensitivity to hydrogen peroxide (H_2_O_2_), with an MIC of 0.5 μg/mL, indicating that oxidative stress effectively eradicates MRSE.

**TABLE 2:** Antibacterial susceptibility testing on MRSE (ATCC35984).

Antimicrobial agents	MIC(μg/mL)	MBC(μg/mL)
Cephalexin	60.0	--
Gentamycin	>500.0	--
Clarithromycin	>500.0	--
Vancomycin	1.2	1.2
OC	240.0	960.0
CA	200.0	800.0
MCA	220.0	860.0
OC+0.01%VC	600.0	--
OC+0.05%VC	>1,000.0	--
H_2_O_2_	0.5	--

“--”indicates that the data is either not applicable or not detected.

### 3.3 OC induces ROS generation on MRSE

**FIGURE 3:**
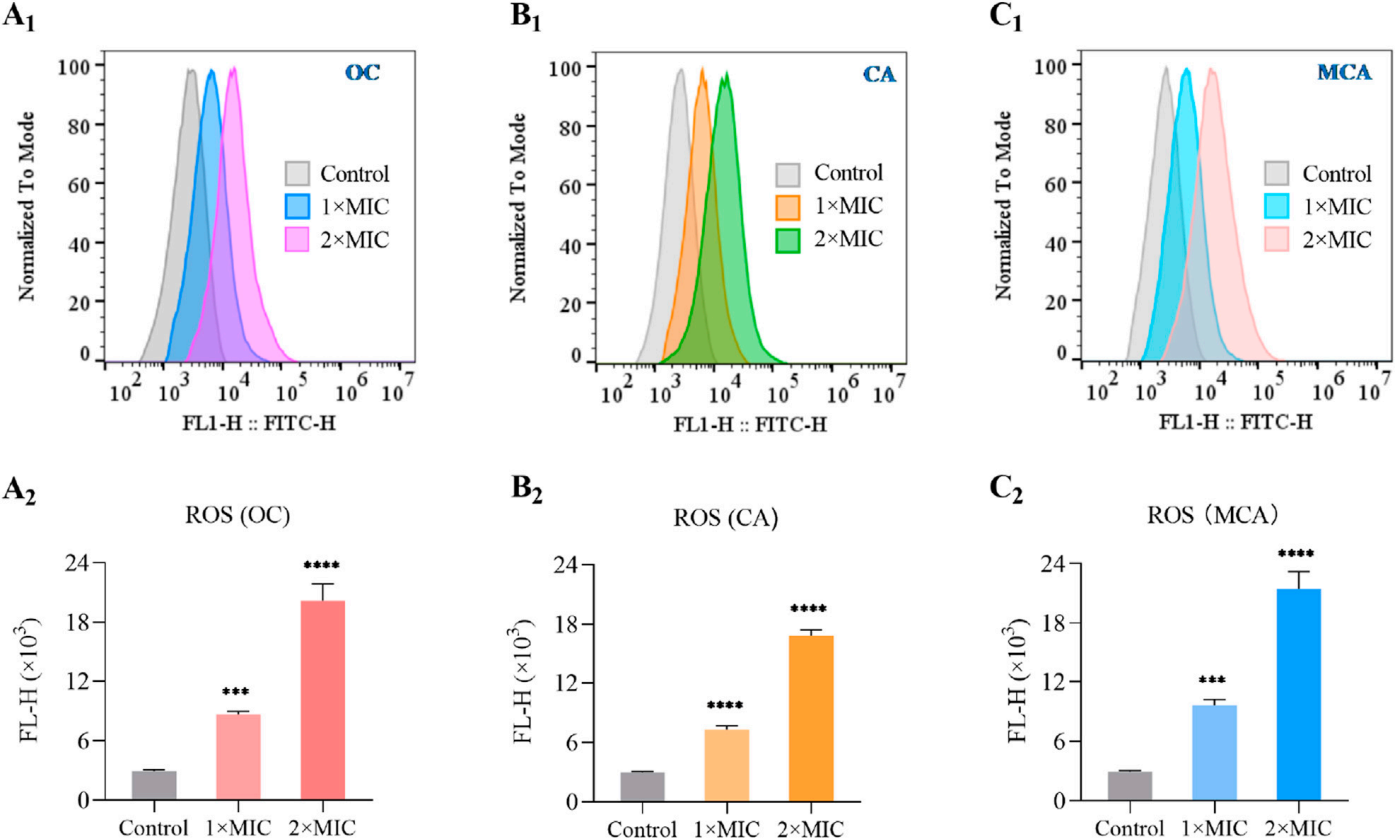
OC induces ROS generation on MRSE. During the logarithmic growth phase, MRSE was exposed to 1×MIC and 2×MIC concentrations of OC for a duration of 4 h, with 0.1% DMSO serving as the control. Intracellular ROS were detected using the CM-H_2_DCFDA probe, and flow cytometry was employed to monitor and generate fluorescence histograms **(A1)**. The medium fluorescence intensity was utilized for quantification **(A2)**. The same method was applied to evaluate CA-induced ROS generation in MRSE, resulting in the generation of fluorescence histograms **(B1)**, with medium fluorescence intensity again used for quantification **(B2)**. Likewise, MCA-induced ROS generation in MRSE was assessed using the same approach, leading to the creation of fluorescence histograms **(C1)**, with medium fluorescence intensity employed for quantification **(C2)**.

As an alternative strategy for the development of novel antibacterial agents, natural products or their derivatives that induce ROS storms are regarded not only as broad-spectrum enhancers of existing antibiotics but also as promising candidates for addressing drug resistance (Dwyer et al., 2009; Van Acker and Coenye, 2017). To evaluate whether OC induces ROS generation in MRSE, we measured the intracellular ROS levels in MRSE exposed to OC, as well as in MRSE cells treated with CA or MCA. As anticipated, after 4 h of exposure to OC at 1×MIC, the ROS concentration increased approximately 2.93-fold compared to the control group. Following 4 h of exposure to OC at 2× MIC, the ROS concentration increased approximately 6.87-fold relative to the control group. OC significantly elevated ROS levels in MRSE, demonstrating a concentration-dependent effect (Figure 3A1,A2). Similarly, treatment with CA resulted in a significant increase in ROS production in MRSE compared to the control group, with a 2.48-fold increase at 1× MIC and a 5.73-fold increase at 2× MIC (Figure 3B1,B2). Likewise, treatment with MCA significantly enhanced ROS production in MRSE relative to the control group, with a 3.27-fold increase at 1× MIC and a 7.31-fold increase at 2× MIC (Figure 3C1,C2). These findings confirm that OC mediates an increase in intracellular ROS in MRSE, primarily induced by CA and MCA. Furthermore, intracellular ROS levels in bacteria serve as a critical indicator of oxidative stress. When ROS levels reach a certain threshold, they can compromise the integrity of the bacterial cell wall, inhibit the transcription and translation of genetic material, and trigger cellular apoptosis.

### 3.4 OC protects RAW264.7 cells from injury induced by MRSE

**FIGURE 4:**
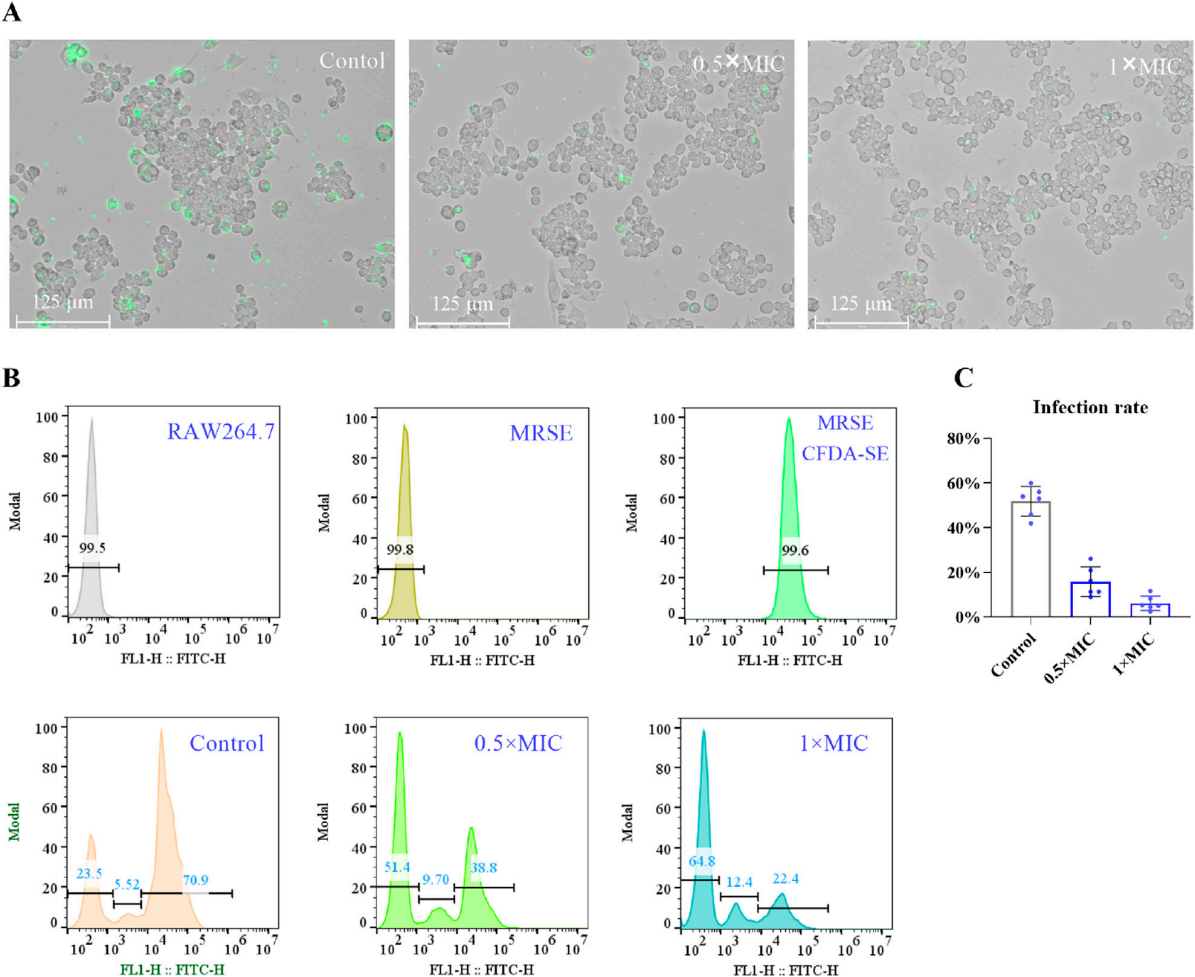
The effect of OC-treated MRSE on autophagy in RAW264.7 macrophages. **(A)** MRSE was treated with OC at 0 and 1×MIC for 4 h, followed by infection with CFDA-SE. RAW264.7 cells were subsequently exposed to MRSE at a multiplicity of infection (MOI) of 10:1 for 2 h. The adhesion of MRSE to RAW264.7 cells was observed using a fluorescence microscope with the green fluorescence channel for CFDA-SE. **(B)** A histogram of the fluorescence intensity obtained from flow cytometry was generated. **(C)** The number of viable MRSE cells within RAW264.7 cells and the percentage of RAW264.7 cells were recorded on Mueller-Hinton agar plates after incubation with OC-treated MRSE and RAW264.7 cells.

ROS serve as a crucial mechanism employed by host cells to eliminate pathogens. Bacteria that exhibit tolerance to oxidative stress possess the ability to evade destruction, thereby enhancing their survival against the host immune response. The oxidative stress tolerance of bacteria is intricately linked to their capacity to circumvent immune detection, and targeting the oxidative stress tolerance mechanisms of bacteria may bolster the host’s immune defenses against bacterial infections (Marrakchi et al., 2014; Poole, 2012). It has been demonstrated that OC targets AhpC, thereby compromising bacterial antioxidant systems, including superoxide dismutase (SOD), peroxiredoxin (Prx), and glutamate-cysteine ligase (GCL). This suggests that OC may augment immune cell-mediated eradication of MRSE (Wagle et al., 2019). To investigate whether OC enhances the capacity of immune cells to eliminate MRSE, we utilized macrophages—cells integral to immune functions related to infection resistance—as our experimental model. We assessed the impact of OC on macrophage-mediated killing of MRSE. The results indicated that in the control group, a majority of RAW264.7 cells exhibited MRSE attachment. However, treatment with OC at 0.5×MIC significantly diminished MRSE adhesion to RAW264.7 cells. At 1×MIC, only a minimal quantity of MRSE remained attached to RAW264.7 cells, indicating that OC effectively reduced MRSE adhesion (Figure 4A). Subsequently, we quantified the number of MRSE-infected RAW264.7 cells using flow cytometry. The flow cytometry histogram revealed that 99.6% of MRSE was labeled with CFDA-SE (Figure 4B), with data collected within the detection region (10^2^–10^3^), which is distinct from the RAW264.7 cells. In the control group, 76.42% of RAW264.7 cells were infected with MRSE, while 5.53% were categorized within the weak fluorescence region. In the 0.5×MIC OC treatment group, 48.5% of RAW264.7 cells were infected with MRSE, with 9.7% in the weak fluorescence region. In the 1×MIC OC treatment group, 38.4% of RAW264.7 cells were infected with MRSE, and 12.4% were in the weak fluorescence region. Notably, compared to the control group, the proportion of MRSE-infected RAW264.7 cells was significantly reduced in the OC-treated groups, and the percentage of RAW264.7 cells in the weak fluorescence region was markedly increased, indicating that OC enhances the ability of RAW264.7 cells to eliminate MRSE. Furthermore, we documented the viable MRSE cells adhering to RAW264.7 cells using Mueller-Hinton agar plates. The percentage of viable MRSE cells was calculated relative to the total number of RAW264.7 cells (Figure 4C). In the control group, the viable MRSE cells constituted 51.83%, whereas in the 0.5×MIC group, this figure decreased to 14.54%, and in the 1×MIC group, it further declined to 6.24%. These findings provide additional evidence that OC protects RAW264.7 cells from injury induced by MRSE.

### 3.5 The target site of OC-induced ROS

**FIGURE 5:**
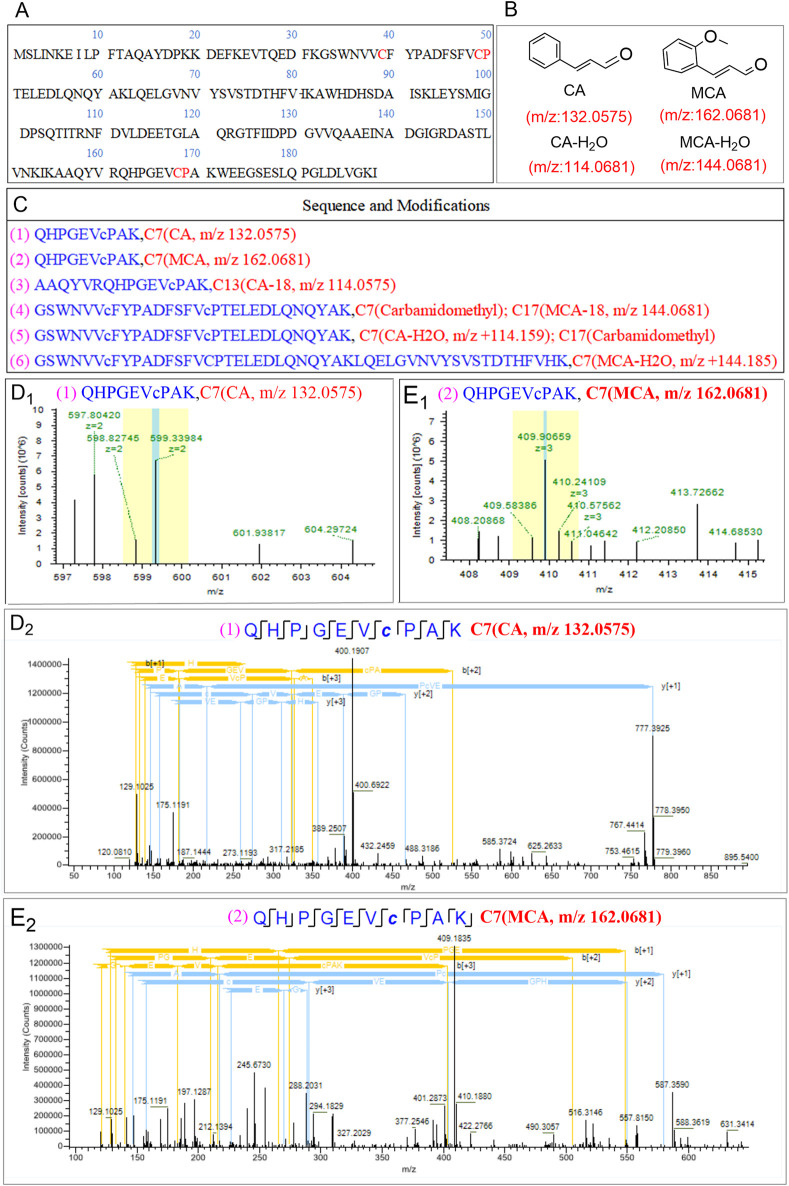
Detection of AhpC binding epitopes covalently modified by OC through modification proteomics. **(A)** Amino acid sequence of the retrieved protein Q5HRY1. **(B)** Mass-to-charge (m/z) values of the main active components of OC: Cinnamaldehyde (CA) and Methoxycinnamaldehyde (MCA). **(C)** Detected representative peptide segments and their modification sites. **(D1, D2)** represents the primary mass spectrum of QHPGEVcPAK (CA Modification at Cysteine Residue C7) peptide segment, and **(E1, E2)** represents peptide consensus view of b, y-ions of the secondary mass spectrum of peptide segment QHPGEVcPAK (MCA Modification at Cysteine Residue C7]).

Modification proteomics enables the direct identification of binding sites between small-molecule compounds and target proteins, thereby facilitating the elucidation of the functions and mechanisms of these proteins in the context of disease. This methodology also aids in the identification of potential therapeutic targets and strategies (Tang et al., 2020). In our investigation of the target of oxidative stress induced by OC in MRSE, we employed modification proteomics to assess the modification status of intracellular proteins in MRSE subjected to OC exposure. Following a 4 h treatment with OC, we extracted intracellular water-soluble proteins, digested them into peptides using trypsin, and analyzed them via Nano-HPLC-MS/MS. The chemical modifications of the peptides were evaluated using the instrument’s software, which revealed modifications on the Q5HRY1 protein (Alkyl hydroperoxide reductase C, AhpC) at Cys39, Cys49, and Cys168, with mass adducts of m/z 132.0575, 114.0575, 162.0681, and 144.0681 (Figure 5A). Notably, these modifications were absent in the control group under identical experimental conditions. The mass adducts associated with Cys39, Cys49, and Cys168 on AhpC were identified as CA, CA- H_2_O_2_, MCA, and MCA- H_2_O_2_ fragments, thereby confirming that CA and MCA in OC covalently modified the AhpC protein (Figure 5B). Figure 5C displays representative peptide segments along with the corresponding modification sites. The results indicated that CA and MCA covalently modified Cys7 with m/z 132.0575 (Figure 5D1,D2) and 162.0681 (Figure 5E1,E2).

AhpC constitutes a significant subtype within the Peroxiredoxin (Prx) family, primarily functioning in concert with its reductive partner protein, Alkyl hydroperoxide reductase F (AhpF), to facilitate the detoxification of hydrogen peroxide and organic peroxides. This enzyme exhibits a broad substrate specificity for peroxides, catalyzing the reduction of hydrogen peroxide and organic hydroperoxides to water and alcohols, thereby playing a crucial protective role against oxidative damage in cellular environments. The active site of AhpC is characterized by conserved redox-active cysteine residues, specifically the peroxidatic cysteine (Cys49, Pro50) and the resolving cysteine (Cys168, Pro169), which participate in nucleophilic attacks on peroxide substrates. The interaction with peroxide results in the oxidation of the Cys (Pro)-SH group, leading to the formation of cysteine sulfenic acid (Cys (Pro)-SOH), which subsequently establishes a disulfide bond with another cysteine residue (-SH). This disulfide bond is later reduced by AhpF, thereby completing the regeneration cycle of AhpC. This typical peroxidatic cysteine (CP) mechanism is integral to the decomposition of hydrogen peroxide. The three conserved cysteines, Cys39, Cys49, and Cys168, within the Q5HRY1 protein are essential for the catalytic mechanism. In the case of OC, CA and MCA covalently modify the catalytic cysteine residues (Cys39, Cys49, and Cys168) of AhpC. The presence of OC inhibits the thiol-specific peroxidase catalytic function of AhpC, thereby disrupting the reduction system and inducing oxidative stress, which is a predictable consequence of this inhibition.

### 3.6 AhpC-protein interactions analysis for DEPs

**FIGURE 6:**
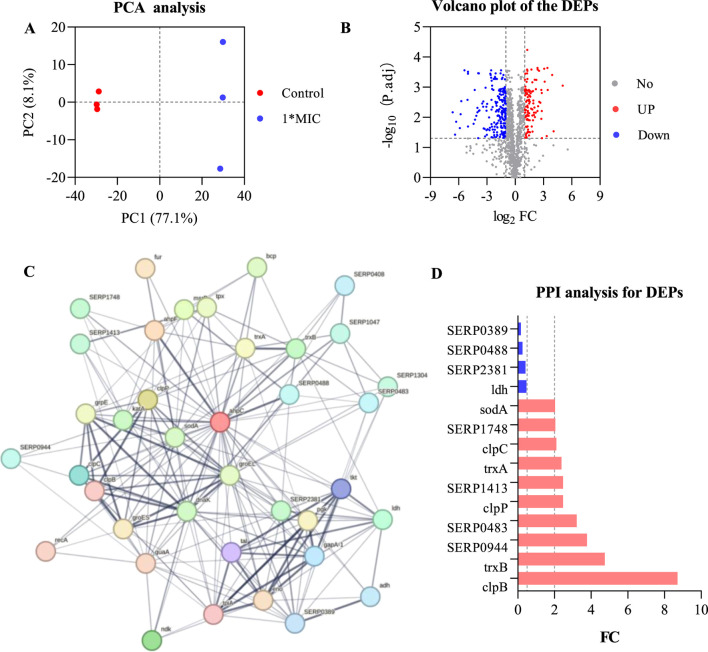
AhpC-Protein interactions analysis for DEPs. **(A)** PCA analysis of the control group and the 1×MIC group. **(B)** Volcano plot analysis of differentially expressed proteins obtained from control and OC-treated samples. **(C)** Analysis of AhpC-protein interactions, with the intensity of the data indicated by line thickness; thicker lines signify a higher confidence in the interaction. Each node in the network represents a protein, while each edge denotes a protein-protein association, with hidden disconnected nodes included in the network. **(D)** Fold change (FC) of dehydrogenases among the differentially expressed proteins.

To analyze the interactions between AhpC (Q5HRY1) and other proteins, and to gain a deeper understanding of its role and regulatory mechanisms within the biological network, we aimed to identify its interacting proteins and downstream signaling pathways, thereby clarifying its functional role in MRSE cells. We employed label-free quantitative proteomics to evaluate differentially expressed proteins (DEPs). The results of Principal Component Analysis (PCA) (Figure 6A) demonstrated a clear separation between the control group and the 1×MIC group (PC1 = 77.1%, PC2 = 8.1%), indicating significant differences between the two groups. The volcano plot analysis of differential proteins (Figure 6B) identified a total of 1,221 proteins (Q-value ≤0.001, P-value <0.05), with 312 proteins exhibiting significant differential expression (P < 0.05), including 199 downregulated proteins and 113 upregulated proteins. We conducted interaction analysis between the target protein AhpC and other proteins identified in the proteomics study, confirming that 28 proteins were closely interacting with AhpC, which included four downregulated proteins and 10 upregulated proteins (Figures 6C,D).

The annotation of differential proteins closely associated with AhpC indicates that the downregulation of lactate dehydrogenase (LDH) compromises lactate metabolism, resulting in a diminished energy supply. This reduction subsequently disrupts the cellular energy balance and exacerbates cellular stress under conditions of oxidative stress. Furthermore, the downregulation of SERP2381 and SERP0488 may interfere with specific stress responses or protein repair mechanisms that remain inadequately understood, thereby further impairing the cell’s capacity to manage oxidative damage. The downregulation of SERP0389 may also influence intracellular metabolic regulation, contributing to an increased instability of the antioxidant system. Among the 10 upregulated proteins, SERP1748 and SERP1413 enhance cellular resistance to oxidative stress by facilitating the repair of oxidative damage and promoting intracellular reduction reactions. Superoxide dismutase A (SodA) mitigates the levels of harmful superoxide anions by converting them into benign hydrogen peroxide and oxygen, thereby alleviating stress on AhpC. Thioredoxin A (TrxA) and Thioredoxin B (TrxB), as critical reductases, restore the functionality of oxidized proteins, thereby enhancing cellular stability in oxidative environments. Additionally, ClpB and ClpP are involved in protein degradation and refolding processes, aiding in the restoration of denatured proteins and the degradation of damaged proteins, which further supports the function of AhpC. Although the precise role of SERP0483 remains unclear, its upregulation may contribute an auxiliary function in the cellular stress response. Meanwhile, ClpC plays a crucial role in ensuring the removal of oxidatively damaged proteins, thereby maintaining the stability of the cellular environment.

AhpC, functioning as a peroxidase, primarily serves to eliminate ROS, such as hydrogen peroxide, thereby preventing oxidative damage to cells. When AhpC’s function is compromised—due to inhibition or energy depletion—it may trigger compensatory mechanisms in MRSE cells, resulting in the overexpression of proteins such as SERP1748, SERP1413, SodA, SERP0944, TrxA, ClpB, SERP0483, ClpP, TrxB, and ClpC. This upregulation enhances the cell’s antioxidant defenses. Conversely, the downregulation of proteins like LDH, SERP2381, SERP0488, and SERP0389 may impair energy metabolism or inhibit cell growth, thereby diminishing the cell’s overall adaptability and increasing the risk of oxidative damage. This response underscores the intricate balance and strategies that MRSE cells employ under oxidative stress. Exposure to OC, which form CA-AhpC or MCA-AhpC complexes, leads to AhpC dysfunction. In response, the cell initiates a series of compensatory reactions to maintain redox balance, resulting in dynamic changes in antioxidant proteins and the downregulation of related proteins, ultimately impacting the cell’s survival and repair capacity.

### 3.7 OC disrupts oxidative stress defense in MRSE, inducing ferroptosis

**FIGURE 7:**
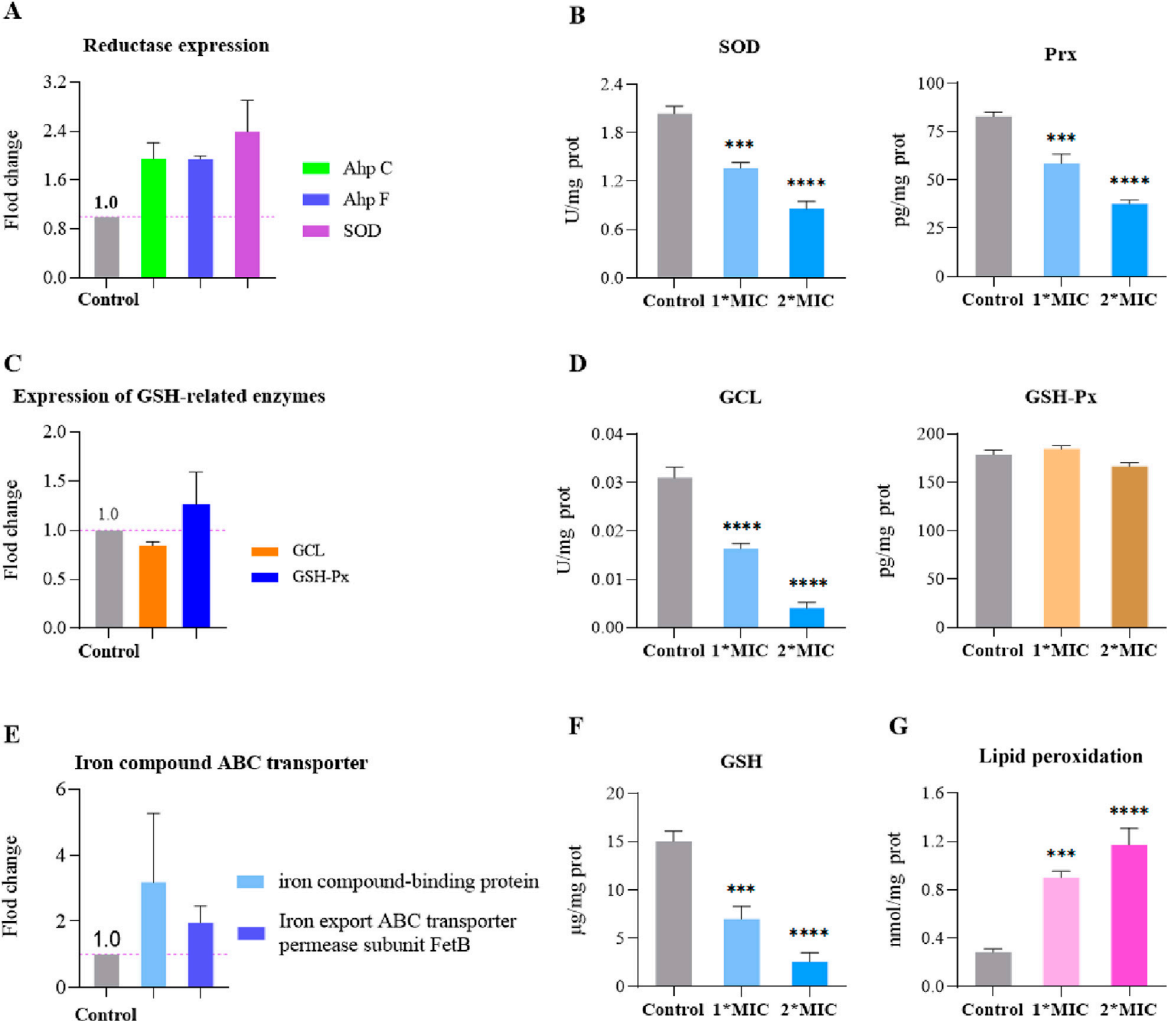
OC disrupts MRSE oxidative stress defense, inducing ferroptosis. MRSE was treated with a specific concentration of OC for 4 h, followed by centrifugation and washing with PBS. After extraction according to the kit instructions, the concentration of bacterial protein was determined using PCA. **(A)** Expression of intracellular ROS detoxification-related reductases, **(B)** Measurement of intracellular superoxide dismutase (SOD) and peroxiredoxin (Prx) activity, **(C)** Expression of glutathione (GSH)-related enzymes, **(D)** Measurement of intracellular GCL and glutathione peroxidase (GSH-Px) activity, **(E)** Expression of iron ion transport-related proteins, **(F)** Measurement of intracellular GSH content, **(G)** Measurement of lipid peroxidation content.

MRSE expresses AhpC, SOD, and GSH, which play crucial roles in detoxifying ROS, enabling the cells to repair or evade oxidative damage caused by these species. To further investigate the molecular mechanism by which OC covalently inhibits AhpC and kills MRSE, we assessed the effects of OC on ROS detoxification and defense, as well as iron ion transport in MRSE. Our findings revealed that exposure to OC upregulated the protein expression of AhpC, AhpF, and SOD in MRSE cells (Figure 7A), indicating that MRSE is attempting to enhance its enzymatic defense system in response to oxidative stress. However, enzyme activity assays demonstrated a significant decrease in the activity of superoxide dismutase (SOD) and peroxiredoxin (Prx) in MRSE cells exposed to OC, exhibiting concentration-dependent effects (Figures 7B,C). When MRSE was treated with OC at 1× MIC, SOD activity was 66.71% of the control group, while Prx activity was 70.68% of the control group. As the OC concentration increased to 2×MIC, SOD activity in MRSE cells decreased to 41.87% of the control group, and Prx activity dropped to 45.61% of the control group. These results, combined with the findings on OC covalently modifying AhpC, confirm that the AhpC-CA or AhpC-MCA complexes formed upon OC exposure stably occupy the catalytic active site of hydrogen peroxide. These complexes cannot be regenerated by AhpF, thereby disrupting the AhpC-AhpF catalytic cycle. Although MRSE upregulated the expression of AhpC and AhpF, which are involved in hydrogen peroxide defense, the sustained inhibition by the covalent inhibitor ultimately leads to the degradation of the enzymatic defense system against ROS in MRSE cells.

Ferroptosis is a form of non-programmed cell death characterized by lipid peroxidation, iron-dependent accumulation, and the collapse of the ROS defense system (Endale et al., 2023). Bacterial ferroptosis involves complex and abnormal biochemical processes, including the metabolism of glutamate and polyunsaturated fatty acids, as well as the biosynthesis of reductive glutathione (GSH), phospholipids, NADPH, and coenzyme Q10 (Wen et al., 2021). The exact mechanism remains unclear; however, it is evident that under conditions where peroxidase detoxification is impaired, the ROS defense system collapses, leading to the depletion of NADPH and GSH. This depletion allows ROS to trigger a series of reaction cascades that culminate in lethal lipid peroxidation, which can induce bacterial ferroptosis (Huang et al., 2023). In our study, we found that MRSE exposed to OC did not exhibit significant changes in the activity of glutathione peroxidase (GSH-Px), an enzyme that utilizes glutathione (GSH) as a substrate to combat oxidative damage. However, the activity of the rate-limiting enzyme GCL, which is responsible for synthesizing GSH, significantly decreased (Figure 7D). This decline led to continuous GSH consumption and a marked increase in lipid peroxidation levels, demonstrating a concentration-dependent effect (Figures 7F,G). When MRSE was treated with 1×MIC of OC, the intracellular reduced GSH content was only 46.63% of that in the control group, while GCL activity was reduced to 45.51% of the control group. The LPO content was approximately three times higher than that of the control group. Upon treatment with 2×MIC of OC, the reduced GSH content in MRSE cells plummeted to 17.28% of the control group, GCL activity decreased to 13.02% of the control group, and the LPO content was roughly four times greater than that of the control group. In MRSE, GSH-Px catalyzes the oxidation of toxic peroxides using GSH, resulting in the formation of harmless hydroxylated compounds and oxidized glutathione (GSSG), thereby protecting the cells from lipid peroxidation damage (Baker and Cohen, 1984). The synthesis rate of the antioxidant substrate GSH is largely dependent on GCL activity, which regulates the rate of GSH synthesis and the GSH/GSSG ratio. The GSH produced subsequently inhibits GCL activity through a feedback mechanism. Under OC stress, the consumption of GSH in MRSE does not stimulate GCL activity, and GSH-Px is unable to obtain the necessary substrate, leading to the accumulation of toxic peroxide compounds that induce LPO damage. The covalent modification of AhpC by OC impairs the peroxidase detoxification process of the AhpC-AhpF system, disrupting both the ROS enzyme defense system and the GSH defense system. Consequently, ROS triggers a series of cascading reactions that result in lethal lipid peroxidation. Furthermore, the upregulation of iron compound-binding proteins and the iron export ABC transporter permease subunit FetB (Figure 7E) suggests that bacterial cells may enter a ferroptosis program.

### 3.8 Metabolic regulation of MRSE cells under oxidative stress

Bacteria enhance their resistance to antibiotics by regulating various metabolic pathways in environments characterized by oxidative stress. Through metabolic regulation, they adapt to external antibiotic pressures, which can influence both the efficacy and toxicity of these drugs (Wang and Sun, 2023) Typically, bacterial metabolic reprogramming is primarily reflected in key pathways such as glycolysis, the tricarboxylic acid (TCA) cycle, and fatty acid metabolism. Understanding these metabolic regulations can provide insights into how bacteria adapt to antibiotic pressure, thereby facilitating the development of new therapeutic strategies against drug-resistant bacteria (Abdelwahed et al., 2022).

**FIGURE 8:**
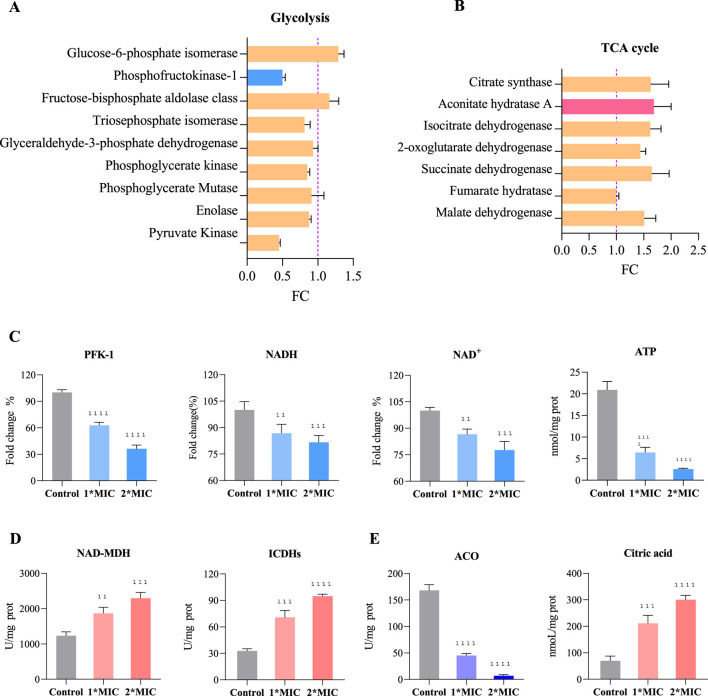
Regulation of Glycolysis and the TCA Cycle in MRSE to Counter OC-Mediated Oxidative Stress. Exposure of MRSE to 1×MIC of OC: **(A)** Expression levels of glycolysis-related enzymes in MRSE, **(B)** Expression levels of TCA cycle-related enzymes in MRSE. Exposure of MRSE to 1×MIC and 2×MIC of OC: **(C)** Activity of the PFK-1 enzyme and intracellular levels of NADH, NAD^+^, and ATP in MRSE, **(D)** Activity of NAD-MDH and ICDHs enzymes in MRSE cells, **(E)** Activity of the ACO enzyme and citrate content in MRSE cells.

Under oxidative stress, bacteria typically accelerate the glycolysis pathway to rapidly generate ATP and metabolic intermediates, enabling them to cope with excessive ROS accumulation and cellular damage, thereby maintaining essential physiological functions (Zhai et al., 2020). However, we observed that exposure to 1×MIC of OC resulted in the upregulation of glucose-6-phosphate isomerase and aldolase expression in MRSE cells, while the expression of key glycolytic enzymes, such as hexokinase, triose phosphate isomerase, glyceraldehyde-3-phosphate dehydrogenase, phosphoglycerate kinase, phosphoglycerate mutase, enolase, and pyruvate kinase, was downregulated (Figure 8A). The activity of hexokinase, a crucial rate-limiting enzyme in glycolysis, significantly decreased, and the levels of NADH, NAD^+^, and ATP within the cells markedly dropped in a concentration-dependent manner (Figure 8C). This suggests that MRSE cells, when confronted with OC-induced oxidative stress, require a substantial amount of NADPH to counteract ROS-induced damage, with the pentose phosphate pathway serving as the primary source of NADPH. The upregulation of glucose-6-phosphate isomerase facilitates a greater metabolic flow into the pentose phosphate pathway, thereby supporting antioxidant responses (Qian et al., 2022). By downregulating the expression or activity of downstream glycolytic enzymes, MRSE cells limit ATP production, prioritizing cellular repair and antioxidant requirements. This metabolic regulation not only mitigates additional ROS damage caused by excessive ATP generation but also enhances bacterial survival under stress conditions. Furthermore, OC exposure influences intracellular NAD^+^ and NADH levels, prompting bacteria to downregulate certain key enzymes to prevent NAD^+^ depletion, thus maintaining the balance of energy metabolism and cell survival. This comprehensive regulation reflects the bacteria’s adaptive response to excessive ROS accumulation under oxidative stress, revealing alterations in their glycolytic metabolism and the strategies they employ to survive and cope with oxidative stress.

The TCA cycle is not only central to cellular energy metabolism but also plays a crucial role in enabling cells to cope with oxidative stress through various compensatory mechanisms. These mechanisms include the generation of NADPH, regulation of ROS production, activation of alternative metabolic pathways, and promotion of mitochondrial autophagy. Such processes ensure that cells maintain metabolic homeostasis under oxidative stress while minimizing ROS-induced damage (Thomas et al., 2013). In response to oxidative stress mediated by OC, MRSE upregulates the expression of several TCA cycle enzymes, including citrate synthase, aconitate hydratase A (ACO), isocitrate dehydrogenases (ICDHs), 2-oxoglutarate dehydrogenase, succinate dehydrogenase, and malate dehydrogenase (NAD-MDH) (Figure 8B). Notably, the activity of key enzymes such as ICDHs and NAD-MDH is significantly increased, exhibiting a concentration-dependent effect (Figure 8D). This suggests that MRSE modulates the flux of the TCA cycle to optimize energy production and the generation of reducing equivalents (NADH and FADH2) in support of intracellular antioxidant reactions. The increased synthesis of intermediates such as α-ketoglutarate and oxaloacetate promote the production of amino acids, fatty acids, and other essential biomolecules, thereby enhancing the cell’s antioxidant capacity and repair mechanisms. Conversely, the conversion of citrate (CA) to isocitrate by aconitate hydratase (ACO) involves a crucial redox-active iron-sulfur cluster [4Fe-4S]. This coordination is mediated by a fourth, unstable iron atom (Fea), which, in the context of oxidative stress, is susceptible to oxidation by ROS. This oxidation transforms the cluster into a [3Fe-4S] state, leading to enzyme inactivation (Mansilla et al., 2023). Exposure to OC significantly decreases ACO activity, resulting in the accumulation of CA within the cell in a concentration-dependent manner (Figure 8E). While MRSE enhances TCA cycle flux to cope with OC-mediated oxidative stress, the inactivation of ACO limits TCA cycle function. The reduction in ACO activity restricts the overall flow of the TCA cycle, adversely affecting ATP and key metabolite production. This limitation in enzyme activity necessitates that bacteria compensate for energy and metabolite deficits through alternative metabolic pathways, such as enhancing glycolysis or utilizing intermediary metabolites. Therefore, bacteria under oxidative stress must not only regulate the metabolic flux of the TCA cycle but also adapt flexibly to the challenges posed by ACO inhibition to maintain physiological function and ensure survival.

**FIGURE 9:**
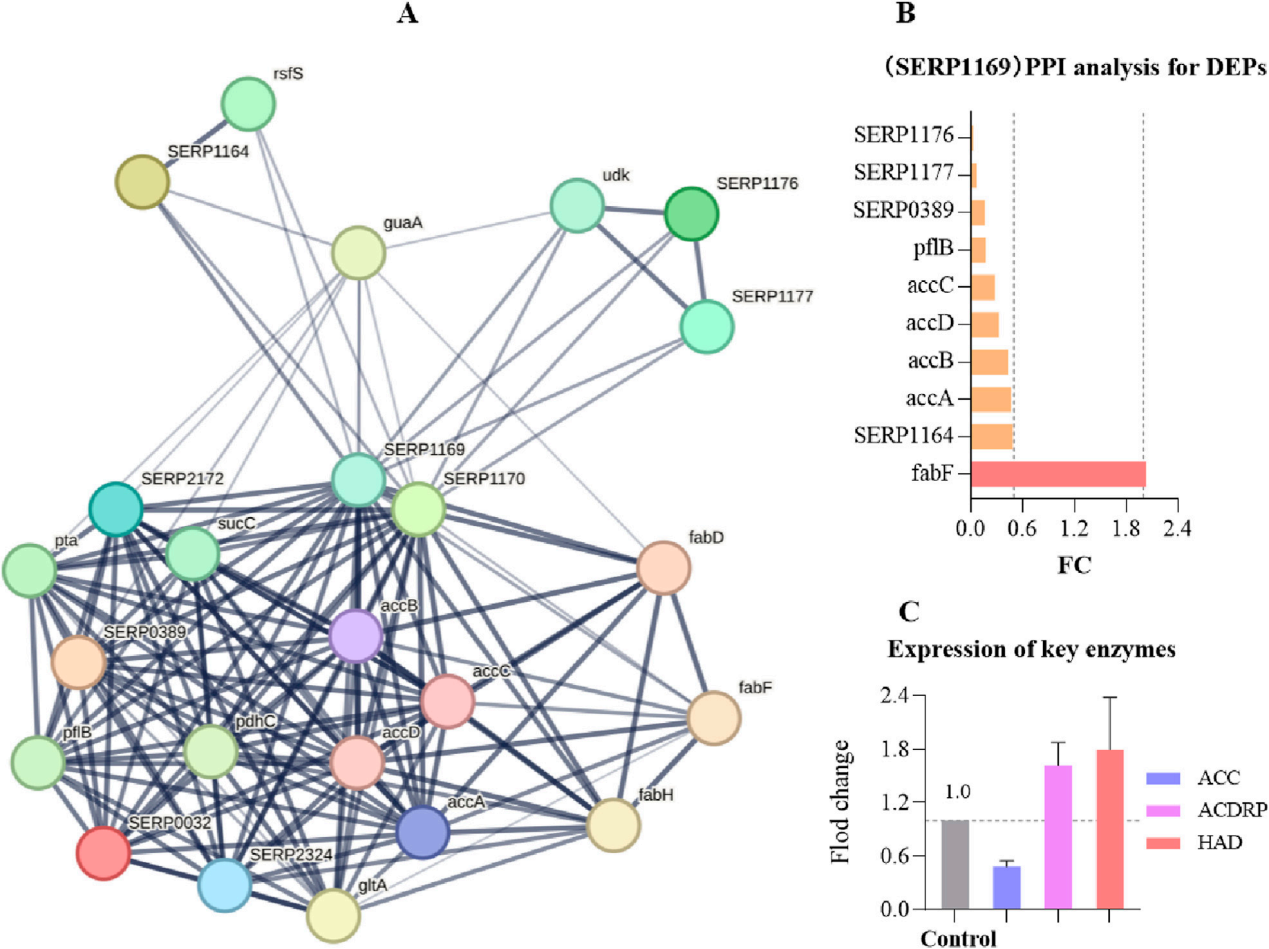
Protein interaction analysis focused on SERP1169. **(A)** SERP1169-protein interaction analysis: The strength of data support is indicated by the thickness of the lines, with thicker lines representing greater confidence in the interactions. Each node in the network represents a protein, while each edge signifies protein-protein associations. **(B)** Significant Differential Proteins in the SERP1169- protein interactions analysis. **(C)** Expression of key fatty acid enzymes.

Protein-protein interaction (PPI) can reveal how proteins cooperate inside and outside the cell to perform various biological functions. By analyzing these interactions, we can better understand the complex signaling pathways, metabolic pathways, and other key biological processes within cells. Acetyl-CoA Carboxylase (ACC) is a crucial enzyme in fatty acid metabolism, catalyzing the conversion of acetyl-CoA to malonyl-CoA, which is a rate-limiting step in fatty acid biosynthesis. The activity of ACC directly influences the rate of fatty acid synthesis, playing a vital role in cellular energy storage and the composition of membrane lipids. In a protein interaction analysis focused on SERP1169, which expresses ACC, and involving 1,221 proteins identified through proteomics, we discovered that SERP1169 directly interacts with 23 proteins (Figure 9A). Exposure to OC resulted in significant downregulation of proteins associated with genes directly linked to SERP1169, including SERP1176, SERP1177, SERP0389, pflB, accC, accD, accB, and SERP1164, while proteins associated with the expression of the fabF gene were significantly upregulated (Figure 9B). This expression pattern reflects the metabolic adaptability of MRSE cells in response to oxidative stress. The downregulated genes are primarily involved in fatty acid synthesis and energy metabolism, particularly enzymes critical to cell membrane composition and function. This may lead to reduced membrane stability, thereby weakening the cell’s resistance to oxidants. Conversely, the upregulation of fabF enhances the synthesis of specific long-chain saturated fatty acids, which may improve membrane fluidity and stability. Furthermore, exposure to 1×MIC of OC resulted in a decrease in the levels of the ACC enzyme, while key enzymes involved in fatty acid metabolism, such as Acyl-CoA Dehydrogenase-Related Protein (ACDRP) and Hydroxyacyl-CoA Dehydrogenase (HAD), were upregulated (Figure 9C). In response to OC stress, MRSE reduces fatty acid synthesis to prevent excessive lipid accumulation and potential lipid peroxidation damage under stress conditions, while simultaneously upregulating enzymes involved in fatty acid degradation to promote the breakdown of fatty acids. This regulation of fatty acid metabolism reveals MRSE’s strategy to adapt to OC-induced oxidative stress, effectively reconstructing a fatty acid metabolic pathway that is favorable for survival. This process not only maintains the integrity of the cell membrane by preventing oxidative damage from excessive lipids but also generates energy that aids in activating intracellular antioxidant defense mechanisms.

## 4 Discussion

Most antibiotics induce the production of ROS in bacteria, particularly those targeting DNA and the cell wall, which can lead to a certain degree of oxidative stress damage. Bacteria that exhibit greater tolerance to oxidative stress may be better equipped to maintain their internal environment, thereby indirectly enhancing their resistance to antibiotics (Vaishampayan and Grohmann, 2022). Multi-drug-resistant MRSE possesses several antioxidant enzymes capable of decomposing ROS, including typical superoxide dismutase (SOD) enzymes, the AhpC-AhpF peroxidase clearance system, and non-enzymatic antioxidants such as glutathione (GSH) and vitamin E. Among these, GSH plays a critical role in ROS clearance through the glutathione peroxidase (GPX)-catalyzed detoxification reaction (Liu, 2009). The ability of GSH to eliminate ROS is contingent upon its biosynthesis. SOD converts superoxide anions into hydrogen peroxide, which is subsequently cleared by peroxiredoxins (Prx). AhpC, an important subtype of the Prx family, can directly convert peroxide substrates into water or corresponding alcohols, thus functioning as a detoxifying agent. AhpF serves as a reductive partner protein for AhpC, specifically catalyzing the transfer of electrons from NADH to AhpC, thereby regenerating AhpC. Strains with a knocked-out AhpC gene exhibit heightened sensitivity to organic hydroperoxides, including hydrogen peroxide (H_2_O_2_) and peroxynitrite (ONOO-) (Wang et al., 2004). Additionally, AhpC plays a crucial role in immune evasion mechanisms and is abundant in MRSE (Wang et al., 2004). Despite the significance of AhpC in bacterial physiology, few studies have focused on utilizing natural compounds as inhibitors of this enzyme.

Our study confirms that OC, derived from the Lauraceae plant genus *Cinnamomum cassia* Presl, and its primary components can mediate the excessive accumulation of ROS within MRSE cells. This accumulation causes oxidative stress-induced bacterial damage and inhibits bacterial adhesion to RAW264.7 cells. Mechanistic studies demonstrate that the main components of OC, CA and MCA, can covalently modify AhpC to form adducts that cannot be regenerated by AhpF. OC disrupts the AhpC-AhpF redox cycle, thereby impairing MRSE’s ability to eliminate peroxides, which leads to sustained ROS accumulation and intracellular oxidative stress.

**FIGURE 10:**
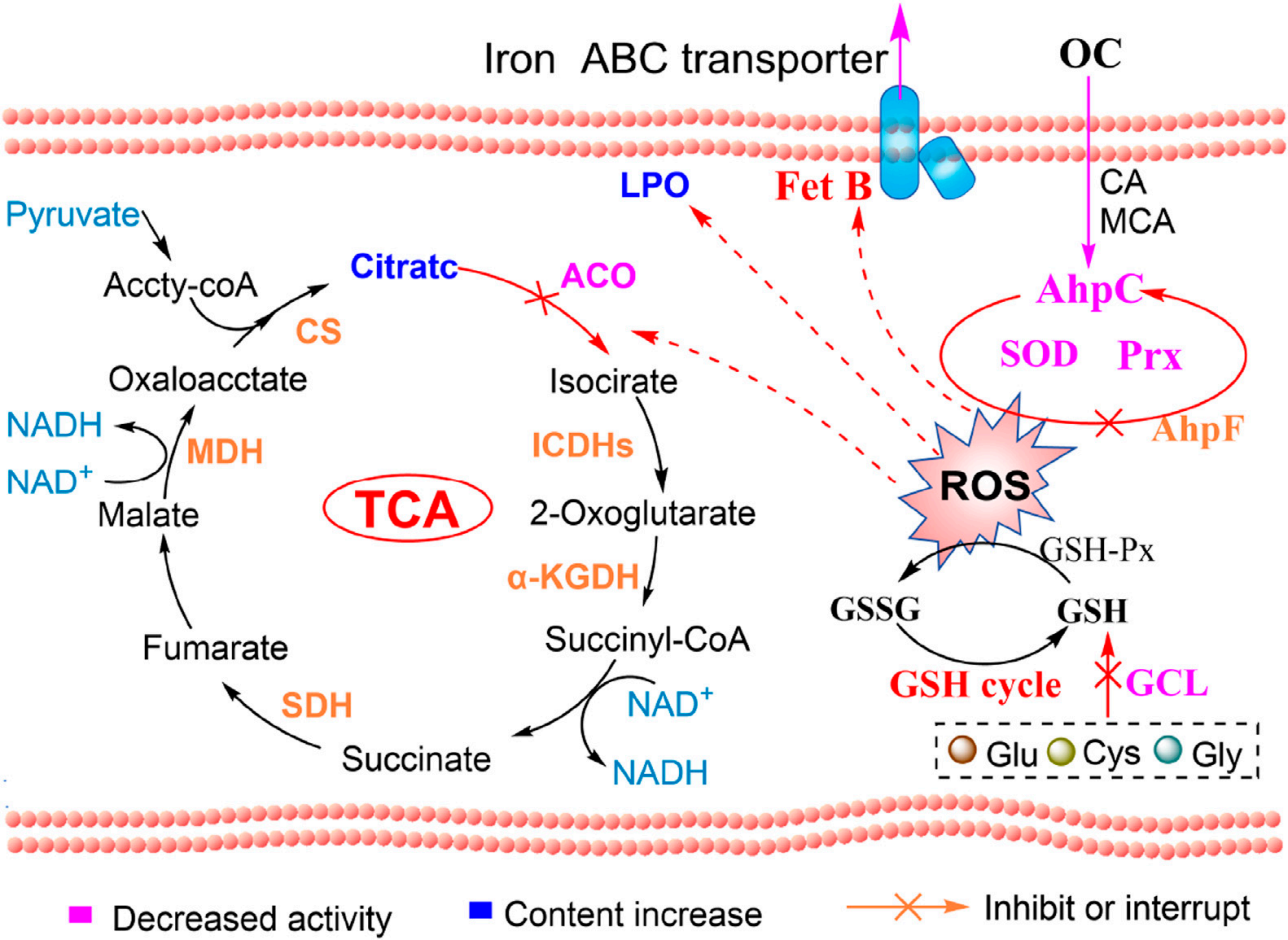
OC-covalent modification of AhpC induces ROS and ferroptosis in MRSE: mechanistic diagram.

In response to oxidative stress, MRSE upregulates the expression of AhpC, AhpF, and superoxide dismutase (SOD), while reprogramming the expression of proteins involved in glycolysis, the tricarboxylic acid cycle (TCA cycle), and fatty acid metabolism. This metabolic reprogramming enhances ROS defense and repairs damage induced by oxidative stress, contributing to resistance against such stress. Due to the sustained covalent inhibition of AhpC, both enzyme and non-enzyme ROS defense systems collapse, ultimately resulting in a trapped ROS storm within MRSE. Under oxidative stress, MRSE reduces the expression of the key rate-limiting enzyme phosphofructokinase-1 (PFK-1) and downstream glycolytic enzymes, while upregulating glucose-6-phosphate isomerase expression. This alteration limits glycolysis and redirects metabolic flux into the pentose phosphate pathway. Although MRSE upregulates the expression of TCA cycle enzymes, the [4Fe-4S] center of aconitase (ACO) is oxidized by ROS, leading to its inactivation. Consequently, citrate cannot be effectively converted to isocitrate, which limits the flux through the TCA cycle. Additionally, MRSE downregulates the expression of the rate-limiting enzyme acetyl-CoA carboxylase (ACC) in fatty acid synthesis and upregulates key enzymes in fatty acid metabolism, such as acyl-CoA dehydrogenase-related protein (ACDRP) and hydroxyacyl-CoA dehydrogenase (HAD). This regulation suppresses fatty acid biosynthesis while enhancing fatty acid metabolism. These metabolic adjustments illustrate MRSE’s response to OC-mediated oxidative stress, primarily reconstructing a metabolic pathway that favors the enhancement of the enzyme defense system. This process supports the biosynthesis and regeneration of reductases or coenzymes involved in ROS detoxification, while simultaneously limiting biological pathways, including ATP production, to prevent excessive ROS generation (Qian et al., 2022; Sun et al., 2025). This metabolic shift initiates the intracellular antioxidant defense mechanism. However, the sustained ROS trapping induced by OC leads to the inactivation of SOD and peroxiredoxin (Prx) enzymes, depletion of glutathione (GSH), lipid peroxidation, and ultimately prompts MRSE to initiate ferroptosis (Figure 10).

In addition, numerous studies have demonstrated that *Oleum Cinnamomi* possesses multifaceted protective effects against MRSE-induced cell damage in macrophages (Damasceno et al., 2025; Guo et al., 2024). These protective mechanisms include antibacterial activity, anti-inflammatory properties, and immune regulation (Rad et al., 2022; Tung et al., 2010). Specifically, *Oleum Cinnamomi* can inhibit the adhesion of MRSE to macrophages, thereby reducing the infection burden (Magetsari PhD et al., 2014). It also enhances the immune function of macrophages by boosting their ability to engulf and clear pathogens. For instance, *Oleum Cinnamomi* has been shown to covalently modify the cysteine residues (Cys248 and Cys249) of the glutamate-cysteine ligase Catalytic Subunit (GCLC) in macrophages through a compound known as CA. This modification enhances the catalytic activity of GCL or GCLC, promoting the synthesis of glutathione, which in turn inhibits excessive inflammatory responses and prevents overactivation of the immune system (Jin et al., 2025). Moreover, research has indicated that *Oleum Cinnamomi* can inhibit MRSE-induced cell apoptosis by modulating the expression of apoptosis-related proteins such as Bcl-2 and Bax. This action further strengthens the immune function of macrophages and enhances their capacity to respond to MRSE infections (Aumeeruddy-Elalfi et al., 2018). In summary, *Oleum Cinnamomi* not only induces MRSE ferroptosis by targeting the AhpC protein but also provides comprehensive protection to macrophages through its antibacterial, anti-inflammatory, antioxidant, and immune-regulatory properties. This enables macrophages to combat MRSE infections more effectively and mitigate cell damage. The multifaceted mechanism of action of *Oleum Cinnamomi* offers a robust scientific rationale for its potential use as an anti-infective adjuvant therapy.

OC, derived from *Cinnamomum cassia* Presl, contains conjugated structures with α, β-unsaturated aldehydes, such as CA and MCA, which can covalently react with the cysteine catalytic residue (-SH) of AhpC. These compounds exert inhibitory effects on peroxiredoxin (Prx), inducing intracellular oxidative stress that can lead to the death of MRSE, thereby representing an important therapeutic strategy against MRSE infections (Jin et al., 2025). The covalent modification of AhpC, which results in ferroptosis in MRSE, is a particularly promising finding, as AhpC plays a crucial role in MRSE’s ROS defense and immune evasion. In conclusion, our study demonstrates that natural products containing conjugated structures with α, β-unsaturated aldehydes can covalently modify AhpC, inhibiting its activity and exhibiting distinct characteristics compared to other identified Prx inhibitors. This contribution to the development of molecules that can inhibit bacterial Prx facilitates the selection of natural products, or the synthesis of inhibitors aimed at enhancing specificity and inhibitory activity against Prx, thereby combating infectious and genetic diseases.

## 5 Conclusion and outlook

CA and its derivative, MCA, derived from the cinnamon, possess conjugated structures containing α,β-unsaturated aldehydes. These compounds can covalently react with the thiol group (-SH) of the catalytic cysteine residue of AhpC, thereby inhibiting AhpC activity. This inhibition induces intracellular oxidative stress, leading to the killing of MRSE. This represents a significant therapeutic strategy against MRSE infections. The covalent modification of AhpC by CA and MCA induces ferroptosis in MRSE, which is a highly positive finding. AhpC is directly involved in MRSE’s defense against ROS and immune evasion, both of which are associated with hospital-acquired infections and high mortality rates. Our research findings demonstrate that natural products containing conjugated α,β-unsaturated aldehydes can covalently modify AhpC and inhibit its activity, exhibiting distinct characteristics from other identified AhpC inhibitors. The ability of these molecules to inhibit bacterial Prx can potentially guide the selection of natural products and even the synthesis of inhibitors aimed at enhancing specificity and inhibitory activity against Prx, thereby combating infectious and hereditary diseases.

In the global health domain, the issue of antibiotic resistance among pathogens is becoming increasingly severe, posing an urgent challenge that demands immediate attention. This problem has spurred scientists to accelerate the development of novel antimicrobial agents. OC and its major component, cinnamaldehyde, have garnered significant attention due to their broad-spectrum antimicrobial activity, low toxicity, and multiple mechanisms of action. However, limitations such as insufficient efficacy and poor water solubility still exist in practical applications. Future research will focus on the design and synthesis of novel CA derivatives. By introducing different functional groups or modifying the molecular structure, it may be possible to enhance their antimicrobial activity, improve stability, and even expand their antimicrobial spectrum. Another direction is to combine CA with other antimicrobial drugs (such as quinolones, aminoglycosides, and β-lactams) to design hybrid molecules, which may generate synergistic effects and delay the emergence of resistance. In terms of mechanism research, gene-editing technologies and single-cell sequencing can help elucidate the interactions between CA and bacterial targets. Cryo-electron microscopy can resolve the three-dimensional structure of CA-target protein complexes, providing a basis for rational drug design. However, the lack of continuous passaging experiments with resistant strains and genomic analysis makes it difficult to predict the resistance risk of CA in long-term clinical use. Therefore, future research should particularly focus on resistance mechanisms, especially cross-resistance with existing antibiotics. Combining knockout strains, transgenic animal models, and multi-omics technologies can provide in-depth insights into the multi-target action network, molecular mechanisms, and stress-induced compensatory mechanisms of CA.

## Data Availability

The datasets presented in this study can be found in online repositories. The names of the repository/repositories and accession number(s) can be found below: https://www.uniprot.org/, Q5HRY1.
